# 4-Chloro-*N*-(2-chloro­phen­yl)benzene­sulfonamide

**DOI:** 10.1107/S1600536811010828

**Published:** 2011-03-26

**Authors:** K. Shakuntala, Sabine Foro, B. Thimme Gowda

**Affiliations:** a Department of Chemistry, Mangalore University, Mangalagangotri 574 199, Mangalore, India; b Institute of Materials Science, Darmstadt University of Technology, Petersenstrasse 23, D-64287, Darmstadt, Germany

## Abstract

In the crystal structure of the title compound, C_12_H_9_Cl_2_NO_2_S, the N—C bond in the C—SO_2_—NH—C segment has *gauche* torsions with respect to the S=O bonds. The mol­ecule is twisted at the S atom with an C—SO_2_—NH—C torsion angle of 57.6 (3)°. The N—H bond is *syn* to the *ortho*-chloro group in the anilino benzene ring. The two benzene rings are tilted relative to each other by 84.7 (1)°. The crystal structure features inversion dimers linked by N—H⋯O(S) hydrogen bonds. An intra­molecular N—H⋯Cl hydrogen bond is also observed.

## Related literature

For our study of the effect of substituents on the oxidative strengths of *N*-chloro,*N*-aryl­sulfonamides, see: Gowda & Shetty (2004 ▶), and on the structures of *N*-(ar­yl)-amides, see: Gowda *et al.* (2004 ▶), *N*-(ar­yl)-aryl­sulfonamides, see: Shakuntala *et al.* (2011 ▶) and *N*-(ar­yl)-methane­sulfonamides, see: Gowda *et al.* (2007 ▶).
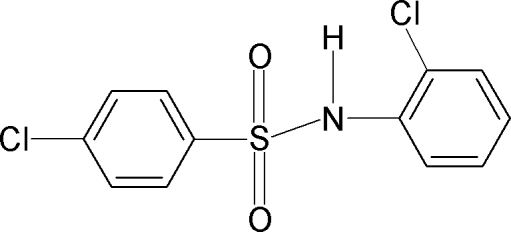


## Experimental

### 

#### Crystal data

C_12_H_9_Cl_2_NO_2_S
*M* *_r_* = 302.16
Monoclinic, 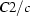

*a* = 14.950 (2) Å
*b* = 12.888 (2) Å
*c* = 14.568 (2) Å
β = 111.41 (1)°
*V* = 2613.2 (6) Å^3^
*Z* = 8
Mo *K*α radiation
μ = 0.65 mm^−1^
*T* = 293 K
0.44 × 0.42 × 0.32 mm

#### Data collection

Oxford Diffraction Xcalibur diffractometer with Sapphire CCD detector
Absorption correction: multi-scan (*CrysAlis RED*; Oxford Diffraction, 2009 ▶) *T*_min_ = 0.764, *T*_max_ = 0.820
5453 measured reflections
2671 independent reflections
2049 reflections with *I* > 2σ(*I*)
*R*_int_ = 0.021

#### Refinement

*R*[*F* ^2^ > 2σ(*F* ^2^)] = 0.042
*wR*(*F* ^2^) = 0.116
*S* = 1.03
2671 reflections
166 parameters
1 restraint
H atoms treated by a mixture of independent and constrained refinement
Δρ_max_ = 0.38 e Å^−3^
Δρ_min_ = −0.38 e Å^−3^

### 

Data collection: *CrysAlis CCD* (Oxford Diffraction, 2009 ▶); cell refinement: *CrysAlis RED* (Oxford Diffraction, 2009 ▶); data reduction: *CrysAlis RED*; program(s) used to solve structure: *SHELXS97* (Sheldrick, 2008 ▶); program(s) used to refine structure: *SHELXL97* (Sheldrick, 2008 ▶); molecular graphics: *PLATON* (Spek, 2009 ▶); software used to prepare material for publication: *SHELXL97*.

## Supplementary Material

Crystal structure: contains datablocks I, global. DOI: 10.1107/S1600536811010828/tk2731sup1.cif

Structure factors: contains datablocks I. DOI: 10.1107/S1600536811010828/tk2731Isup2.hkl

Additional supplementary materials:  crystallographic information; 3D view; checkCIF report

## Figures and Tables

**Table 1:** Hydrogen-bond geometry (Å, °)

*D*—H⋯*A*	*D*—H	H⋯*A*	*D*⋯*A*	*D*—H⋯*A*
N1—H1*N*⋯O2^i^	0.81 (2)	2.29 (2)	3.044 (2)	155 (3)
N1—H1*N*⋯Cl2	0.81 (2)	2.57 (3)	2.945 (2)	110 (2)

Symmetry code: (i) 
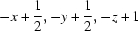
.

**Fig. 1.**
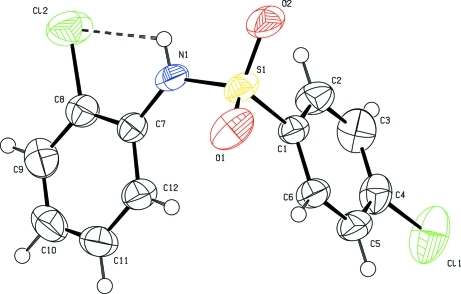
Molecular structure of (I), showing the atom labelling scheme and displacement ellipsoids are drawn at the 50% probability level. Hydrogen bonding shown as dashed lines.

**Fig. 2.**
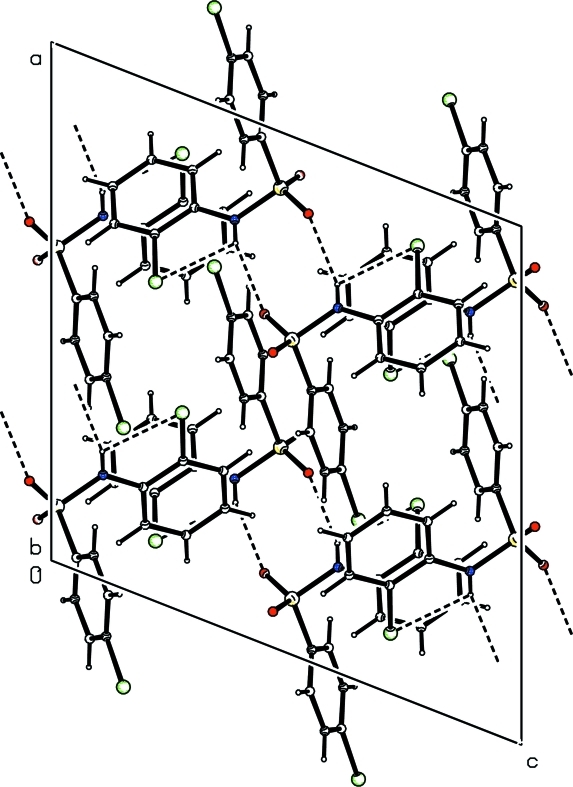
Molecular packing of (I) with hydrogen bonding shown as dashed lines.

## supplementary crystallographic information

### Comment

The sulfonamide moieties are important constituents of many biologically significant compounds. As a part of a study of the substituent effects on the structures and other aspects of this class of compounds (Gowda & Shetty, 2004; Gowda *et al.* 2004, 2007; Shakuntala *et al.*, 2011), in the present work, the crystal structure of 4-chloro-*N*-(2-chlorophenyl)benzenesulfonamide (I) has been determined (Fig. 1). The conformation of the N—C bond in the C—SO_2_—NH—C segment is *gauche* with respect to the S═O bonds. The molecule is bent at the S atom with the C—SO_2_—NH—C torsion angle of 57.6 (3)°, compared to the value of -58.4 (3)° in 4-chloro-*N*-(3-chlorophenyl)benzenesulfonamide (II) (Shakuntala *et al.*, 2011). The conformation of the N—H bond in the C—SO_2_—NH—C segment in (I) is *syn* to the *ortho*-chloro group in the adjacent anilino benzene ring, in contrast to the *anti* conformation observed between the N—H bond and the *meta*-chloro group in the anilino benzene ring of (II). The sulfonyl and the anilino benzene rings in (I) are tilted relative to each other by 84.7 (1)°, compared to the value of 77.1 (1)° in (II).

The structure shows simultaneous N—H···Cl intramolecular and N—H···O intermolecular H-bonding (Table 1). The crystal packing in (I) features dimeric aggregates stabilised by N—H···O(S) hydrogen bonds as shown in Fig.2.

### Experimental

A solution of chlorobenzene (10 ml) in chloroform (40 ml) was treated drop wise with chlorosulfonic acid (25 ml) at 273 K. After the initial evolution of hydrogen chloride subsided, the reaction mixture was brought to room temperature and poured into crushed ice in a beaker. The chloroform layer was separated, washed with cold water and allowed to evaporate slowly. The residual 4-chlorobenzenesulfonylchloride was treated with 2-chloroaniline in a stoichiometric ratio and boiled for ten minutes. The reaction mixture was then cooled to room temperature and added to ice cold water (100 ml). The resultant 4-chloro-*N*-(2-chlorophenyl)benzenesulfonamide was filtered under suction and washed thoroughly with cold water. It was then recrystallized to constant melting point from dilute ethanol.

Colorless prisms used in X-ray diffraction studies were grown in ethanolic solution by slow evaporation at room temperature.

### Refinement

The N-H atom was located in a difference map and refined with the distance restraint N—H = 0.86 ±0.02 Å. The other H atoms were positioned with idealized geometry using a riding model with C—H = 0.93 Å. All H atoms were refined with isotropic displacement parameters set to 1.2 times of the *U*_eq_ of the parent atom.

### Crystal data

**Table:**

C_12_H_9_Cl_2_NO_2_S	*F*(000) = 1232
*M**_r_* = 302.16	*D*_x_ = 1.536 Mg m^−^^3^
Monoclinic, *C*2/*c*	Mo *K*α radiation, λ = 0.71073 Å
Hall symbol: -C 2yc	Cell parameters from 2370 reflections
*a* = 14.950 (2) Å	θ = 2.8–27.7°
*b* = 12.888 (2) Å	µ = 0.65 mm^−^^1^
*c* = 14.568 (2) Å	*T* = 293 K
β = 111.41 (1)°	Prism, colourless
*V* = 2613.2 (6) Å^3^	0.44 × 0.42 × 0.32 mm
*Z* = 8	

### Data collection

**Table:**

Oxford Diffraction Xcalibur diffractometer with Sapphire CCD	2671 independent reflections
Radiation source: fine-focus sealed tube	2049 reflections with *I* > 2σ(*I*)
graphite	*R*_int_ = 0.021
Rotation method data acquisition using ω and phi scans.	θ_max_ = 26.4°, θ_min_ = 2.9°
Absorption correction: multi-scan (*CrysAlis RED*; Oxford Diffraction, 2009)	*h* = −18→13
*T*_min_ = 0.764, *T*_max_ = 0.820	*k* = −16→9
5453 measured reflections	*l* = −18→18

### Refinement

**Table:**

Refinement on *F*^2^	Primary atom site location: structure-invariant direct methods
Least-squares matrix: full	Secondary atom site location: difference Fourier map
*R*[*F*^2^ > 2σ(*F*^2^)] = 0.042	Hydrogen site location: inferred from neighbouring sites
*wR*(*F*^2^) = 0.116	H atoms treated by a mixture of independent and constrained refinement
*S* = 1.03	*w* = 1/[σ^2^(*F*_o_^2^) + (0.0576*P*)^2^ + 2.4691*P*] where *P* = (*F*_o_^2^ + 2*F*_c_^2^)/3
2671 reflections	(Δ/σ)_max_ < 0.001
166 parameters	Δρ_max_ = 0.38 e Å^−^^3^
1 restraint	Δρ_min_ = −0.38 e Å^−^^3^

### Special details

**Table:**

Experimental. Empirical absorption correction using spherical harmonics, implemented in SCALE3 ABSPACK scaling algorithm.
Geometry. All e.s.d.'s (except the e.s.d. in the dihedral angle between two l.s. planes) are estimated using the full covariance matrix. The cell e.s.d.'s are taken into account individually in the estimation of e.s.d.'s in distances, angles and torsion angles; correlations between e.s.d.'s in cell parameters are only used when they are defined by crystal symmetry. An approximate (isotropic) treatment of cell e.s.d.'s is used for estimating e.s.d.'s involving l.s. planes.
Refinement. Refinement of *F*^2^ against ALL reflections. The weighted *R*-factor *wR* and goodness of fit *S* are based on *F*^2^, conventional *R*-factors *R* are based on *F*, with *F* set to zero for negative *F*^2^. The threshold expression of *F*^2^ > σ(*F*^2^) is used only for calculating *R*-factors(gt) *etc*. and is not relevant to the choice of reflections for refinement. *R*-factors based on *F*^2^ are statistically about twice as large as those based on *F*, and *R*- factors based on ALL data will be even larger.

### Fractional atomic coordinates and isotropic or equivalent isotropic displacement parameters (Å^2^)

**Table:**

	*x*	*y*	*z*	*U*_iso_*/*U*_eq_	
C1	0.47546 (14)	0.24207 (18)	0.44604 (15)	0.0382 (5)	
C2	0.45846 (18)	0.3414 (2)	0.4102 (2)	0.0552 (6)	
H2	0.4028	0.3758	0.4071	0.066*	
C3	0.5242 (2)	0.3898 (2)	0.3789 (2)	0.0636 (7)	
H3	0.5138	0.4574	0.3549	0.076*	
C4	0.60507 (18)	0.3372 (2)	0.38344 (18)	0.0538 (6)	
C5	0.62200 (18)	0.2385 (2)	0.4181 (2)	0.0547 (6)	
H5	0.6773	0.2040	0.4202	0.066*	
C6	0.55683 (16)	0.18986 (19)	0.45023 (19)	0.0473 (6)	
H6	0.5678	0.1224	0.4745	0.057*	
C7	0.28930 (15)	0.11084 (18)	0.30448 (17)	0.0418 (5)	
C8	0.20809 (17)	0.12287 (19)	0.21954 (17)	0.0453 (5)	
C9	0.1974 (2)	0.0696 (2)	0.13415 (19)	0.0575 (7)	
H9	0.1418	0.0782	0.0787	0.069*	
C10	0.2680 (2)	0.0043 (2)	0.1304 (2)	0.0650 (8)	
H10	0.2613	−0.0308	0.0725	0.078*	
C11	0.3492 (2)	−0.0086 (2)	0.2135 (2)	0.0616 (7)	
H11	0.3977	−0.0526	0.2113	0.074*	
C12	0.35983 (18)	0.0428 (2)	0.3001 (2)	0.0531 (6)	
H12	0.4146	0.0317	0.3559	0.064*	
N1	0.29597 (13)	0.16494 (18)	0.39002 (15)	0.0509 (5)	
H1N	0.2514 (16)	0.199 (2)	0.392 (2)	0.061*	
O1	0.43100 (13)	0.08327 (17)	0.52862 (14)	0.0651 (5)	
O2	0.36333 (12)	0.25214 (19)	0.54668 (13)	0.0696 (6)	
Cl1	0.68971 (6)	0.39830 (8)	0.34678 (7)	0.0926 (3)	
Cl2	0.11621 (5)	0.20293 (8)	0.22150 (6)	0.0787 (3)	
S1	0.39197 (4)	0.18111 (5)	0.48790 (4)	0.04654 (19)	

### Atomic displacement parameters (Å^2^)

**Table:**

	*U*^11^	*U*^22^	*U*^33^	*U*^12^	*U*^13^	*U*^23^
C1	0.0337 (10)	0.0455 (12)	0.0368 (10)	0.0025 (9)	0.0147 (8)	−0.0003 (9)
C2	0.0461 (13)	0.0534 (15)	0.0642 (16)	0.0149 (12)	0.0178 (12)	0.0087 (13)
C3	0.0659 (17)	0.0492 (16)	0.0667 (17)	0.0025 (13)	0.0134 (14)	0.0208 (13)
C4	0.0533 (14)	0.0599 (16)	0.0506 (14)	−0.0160 (12)	0.0220 (11)	0.0032 (12)
C5	0.0455 (13)	0.0527 (15)	0.0770 (17)	−0.0031 (11)	0.0357 (13)	−0.0058 (13)
C6	0.0410 (12)	0.0394 (12)	0.0684 (15)	0.0019 (10)	0.0280 (11)	0.0019 (11)
C7	0.0399 (11)	0.0424 (12)	0.0509 (13)	−0.0072 (9)	0.0258 (10)	−0.0039 (10)
C8	0.0481 (12)	0.0448 (13)	0.0493 (13)	−0.0039 (10)	0.0254 (10)	0.0020 (11)
C9	0.0653 (16)	0.0621 (17)	0.0493 (14)	−0.0150 (14)	0.0258 (12)	−0.0041 (13)
C10	0.084 (2)	0.0568 (16)	0.0720 (18)	−0.0214 (15)	0.0499 (17)	−0.0228 (14)
C11	0.0672 (17)	0.0485 (15)	0.086 (2)	−0.0042 (13)	0.0478 (16)	−0.0142 (14)
C12	0.0469 (13)	0.0514 (15)	0.0688 (16)	−0.0006 (11)	0.0303 (12)	−0.0044 (13)
N1	0.0315 (9)	0.0706 (14)	0.0533 (11)	0.0050 (9)	0.0188 (9)	−0.0145 (11)
O1	0.0529 (10)	0.0805 (14)	0.0681 (12)	0.0049 (10)	0.0293 (9)	0.0315 (11)
O2	0.0449 (9)	0.1162 (18)	0.0546 (10)	−0.0005 (10)	0.0264 (8)	−0.0272 (11)
Cl1	0.0793 (5)	0.1133 (7)	0.0893 (6)	−0.0409 (5)	0.0355 (5)	0.0226 (5)
Cl2	0.0598 (4)	0.1065 (7)	0.0623 (4)	0.0306 (4)	0.0135 (3)	−0.0056 (4)
S1	0.0338 (3)	0.0699 (4)	0.0412 (3)	0.0022 (3)	0.0200 (2)	0.0005 (3)

### Geometric parameters (Å, °)

**Table:**

C1—C2	1.371 (3)	C7—N1	1.399 (3)
C1—C6	1.372 (3)	C8—C9	1.378 (3)
C1—S1	1.761 (2)	C8—Cl2	1.727 (2)
C2—C3	1.375 (4)	C9—C10	1.367 (4)
C2—H2	0.9300	C9—H9	0.9300
C3—C4	1.367 (4)	C10—C11	1.376 (4)
C3—H3	0.9300	C10—H10	0.9300
C4—C5	1.357 (4)	C11—C12	1.381 (4)
C4—Cl1	1.732 (2)	C11—H11	0.9300
C5—C6	1.376 (3)	C12—H12	0.9300
C5—H5	0.9300	N1—S1	1.625 (2)
C6—H6	0.9300	N1—H1N	0.807 (17)
C7—C8	1.390 (3)	O1—S1	1.425 (2)
C7—C12	1.391 (3)	O2—S1	1.4221 (19)
			
C2—C1—C6	120.9 (2)	C7—C8—Cl2	119.58 (18)
C2—C1—S1	119.47 (17)	C10—C9—C8	120.4 (3)
C6—C1—S1	119.66 (18)	C10—C9—H9	119.8
C1—C2—C3	119.6 (2)	C8—C9—H9	119.8
C1—C2—H2	120.2	C9—C10—C11	119.0 (3)
C3—C2—H2	120.2	C9—C10—H10	120.5
C4—C3—C2	119.0 (2)	C11—C10—H10	120.5
C4—C3—H3	120.5	C10—C11—C12	121.1 (3)
C2—C3—H3	120.5	C10—C11—H11	119.5
C5—C4—C3	121.6 (2)	C12—C11—H11	119.5
C5—C4—Cl1	118.9 (2)	C11—C12—C7	120.5 (3)
C3—C4—Cl1	119.5 (2)	C11—C12—H12	119.7
C4—C5—C6	119.6 (2)	C7—C12—H12	119.7
C4—C5—H5	120.2	C7—N1—S1	126.74 (16)
C6—C5—H5	120.2	C7—N1—H1N	121 (2)
C1—C6—C5	119.2 (2)	S1—N1—H1N	112 (2)
C1—C6—H6	120.4	O2—S1—O1	119.12 (13)
C5—C6—H6	120.4	O2—S1—N1	104.32 (10)
C8—C7—C12	117.4 (2)	O1—S1—N1	110.37 (13)
C8—C7—N1	119.5 (2)	O2—S1—C1	109.18 (12)
C12—C7—N1	123.1 (2)	O1—S1—C1	107.73 (10)
C9—C8—C7	121.6 (2)	N1—S1—C1	105.30 (10)
C9—C8—Cl2	118.8 (2)		
			
C6—C1—C2—C3	−0.6 (4)	C8—C9—C10—C11	1.0 (4)
S1—C1—C2—C3	179.0 (2)	C9—C10—C11—C12	0.4 (4)
C1—C2—C3—C4	0.5 (4)	C10—C11—C12—C7	−1.5 (4)
C2—C3—C4—C5	−0.1 (4)	C8—C7—C12—C11	1.3 (3)
C2—C3—C4—Cl1	−178.6 (2)	N1—C7—C12—C11	179.9 (2)
C3—C4—C5—C6	−0.4 (4)	C8—C7—N1—S1	−165.93 (19)
Cl1—C4—C5—C6	178.1 (2)	C12—C7—N1—S1	15.4 (4)
C2—C1—C6—C5	0.2 (4)	C7—N1—S1—O2	172.5 (2)
S1—C1—C6—C5	−179.4 (2)	C7—N1—S1—O1	−58.4 (2)
C4—C5—C6—C1	0.3 (4)	C7—N1—S1—C1	57.6 (2)
C12—C7—C8—C9	0.1 (3)	C2—C1—S1—O2	−44.3 (2)
N1—C7—C8—C9	−178.7 (2)	C6—C1—S1—O2	135.3 (2)
C12—C7—C8—Cl2	177.84 (18)	C2—C1—S1—O1	−175.0 (2)
N1—C7—C8—Cl2	−0.9 (3)	C6—C1—S1—O1	4.6 (2)
C7—C8—C9—C10	−1.2 (4)	C2—C1—S1—N1	67.2 (2)
Cl2—C8—C9—C10	−179.0 (2)	C6—C1—S1—N1	−113.2 (2)

### Hydrogen-bond geometry (Å, °)

**Table:**

*D*—H···*A*	*D*—H	H···*A*	*D*···*A*	*D*—H···*A*
N1—H1N···O2^i^	0.81 (2)	2.29 (2)	3.044 (2)	155 (3)
N1—H1N···Cl2	0.81 (2)	2.57 (3)	2.945 (2)	110 (2)

Symmetry codes: (i) −*x*+1/2, −*y*+1/2, −*z*+1.
